# Neurological Sequelae of Post-COVID-19 Fatigue: A Narrative Review of Dipeptidyl Peptidase IV-Mediated Cerebrovascular Complications

**DOI:** 10.3390/cimb46120811

**Published:** 2024-11-28

**Authors:** Che Mohd Nasril Che Mohd Nassir, Muhammad Danial Che Ramli, Usman Jaffer, Hafizah Abdul Hamid, Muhammad Zulfadli Mehat, Mazira Mohamad Ghazali, Ebrahim Nangarath Kottakal Cheriya

**Affiliations:** 1 Department of Anatomy and Physiology, Faculty of Medicine, School of Basic Medical Sciences, Universiti Sultan Zainal Abidin, Kuala Terengganu 20400, Terengganu, Malaysia; mazira.mohamadghazali@gmail.com; 2 Faculty of Health and Life Sciences, Management and Science University, Shah Alam 40150, Selangor, Malaysia; 3 Kulliyyah of Islamic Revealed Knowledge and Human Sciences, International Islamic University Malaysia, Kuala Lumpur 50728, Malaysia; jafferu@iium.edu.my; 4 Department of Human Anatomy, Faculty of Medicine and Health Sciences, Universiti Putra Malaysia (UPM), Serdang 43400, Selangor, Malaysia; a_hafizah@upm.edu.my (H.A.H.); m_zulfadli@upm.edu.my (M.Z.M.); 5 Department of Neurosciences, School of Medical Sciences, Universiti Sains Malaysia, Kubang Kerian, Kota Bharu 16150, Kelantan, Malaysia; 6 Department of Physiology, International Medical School (IMS), Management and Science University, Shah Alam 40150, Selangor, Malaysia; ebrahim_nangarath@msu.edu.my

**Keywords:** post-COVID-19 fatigue, neurodegenerative diseases, dipeptidyl peptidase IV, cerebrovascular, SARS-CoV-2

## Abstract

Coronavirus disease 2019 (COVID-19) has been a global pandemic affecting millions of people’s lives, which has led to ‘post-COVID-19 fatigue’. Alarmingly, severe acute respiratory syndrome-coronavirus 2 (SARS-CoV-2) not only infects the lungs but also influences the heart and brain. Endothelial cell dysfunction and hypercoagulation, which we know occur with this infection, lead to thrombo-inflammation that can manifest as many myriad cardio-cerebrovascular disorders, such as brain fog, fatigue, cognitive dysfunction, etc. Additionally, SARS-CoV-2 has been associated with oxidative stress, protein aggregation, cytokine storm, and mitochondrial dysfunction in neurodegenerative diseases. Accordingly, the identification of molecular targets involved in these actions could provide strategies for preventing and treating this disease. In particular, the very common enzyme dipeptidyl peptidase IV (DPPIV) has recently been identified as a candidate co-receptor for the cell entry of the SARS-CoV-2 virus with its involvement in infection. In addition, DPPIV has been reported as a co-receptor for some viruses such as Middle East respiratory syndrome-coronavirus (MERS-CoV). It mediates immunologic reactions and diseases such as type 2 diabetes mellitus, obesity, and hypertension, which have been considered the prime risk factors for stroke among other types of cardio-cerebrovascular diseases. Unlike angiotensin-converting enzyme 2 (ACE2), DPPIV has been implicated in aggravating the course of infection due to its disruptive effect on inflammatory signaling networks and the neuro–glia–vascular unit. Regarding the neurological, physiological, and molecular grounds governing post-COVID-19 fatigue, this review focuses on DPPIV as one of such reasons that progressively establishes cerebrovascular grievances following SARS-CoV infection.

## 1. Introduction

Public worry is still raised by the long-lasting physical and mental health problems that have resulted from severe acute respiratory syndrome-coronavirus 2 (SARS-CoV-2) infection. The COVID-19 pandemic’s worldwide effects include aspects related to sociopsychology, health, and the economy [1,2,3]. Many of the patients who have recovered from coronavirus disease 2019 (COVID-19) have been described as having symptoms that continue for several months after they first became ill. Long COVID, post-COVID-19 syndrome, chronic COVID-19 syndrome, post-acute sequelae of COVID-19 (PASC), or post-COVID-19 fatigue are some names for this illness [4,5,6]. However, this manuscript will adopt the term “post-COVID-19 fatigue”.

Alarmingly, post-COVID-19 fatigue is not limited to elderly patients with severe illness; younger generations with mild-to-moderate symptoms are also affected. Moreover, more than half of young adults have reported symptoms that last longer than six months, suggesting that long-term physical and mental repercussions might occur even in cases of mild-to-moderate illness [7,8,9]. Many theories concerning the duration of symptoms after SARS-CoV-2 infection are being offered by new research. Additionally, post-COVID-19 fatigue is thought to be caused by the immune system imbalance and residual virus particles in the body. Results showing that SARS-CoV-2 ribonucleic acid (RNA) was still present in COVID-19-infected people’s excretions several months after infection lend support to this. Moreover, studies have shown evidence of a viral presence in several tissues, including the brain [10,11]. Moreover, patients with chronic autoimmune diseases also experience persistent symptoms associated with post-COVID-19 fatigue [12]. The probability or intensity of long-term symptoms does not seem to be correlated with the severity of the initial illness [13].

In addition to the pathological consequences, lockdowns, and the increasing use of remote work have had a profound impact on socio-economic dynamics and face-to-face contact. These changes have introduced temporal distortions, likely contributing to prolonged psychological and physiological disruptions [14,15]. Nutritional modifications and a reduction in daily physical activity may potentially have a substantial impact on the results of post-COVID-19 fatigue [16]. It is becoming more and more clear that post-COVID-19 fatigue may trigger a secondary public health emergency, especially when it comes to cerebrovascular illnesses including depression, stroke, and cognitive decline [17,18].

Moreover, multiple reports have shown that the COVID-19 pandemic’s isolation and lockdown protocols have harmed children’s emotional and physical health. While many young people and children infected with SARS-CoV-2 have moderate disease or show no symptoms at all, new research suggests that, in the months after infection, 5–10% of all children may show new symptoms, including neurological and neurocognitive sequelae [19,20]. Owing to the wide range of symptoms linked to COVID-19, there is no specific treatment option available at this time. Although this topic needs further research through additional studies, it is fortunate that most children with post-COVID-19 fatigue tend to recover faster than most adults. There is currently a great deal of clinical and basic research being carried out to try and understand the processes behind post-COVID-19 fatigue produced by SARS-CoV-2, even though the possible mechanisms of vascular and neurological involvement during SARS and MERS infections have been largely understood. Further research is needed to improve our capacity to predict and mitigate the impact of post-infectious sequelae, specifically on cerebrovascular outcomes. The fact that post-COVID-19 fatigue is a complex illness involving multiple organs, cell types, and chemicals highlights the significance of thorough research in this field.

Thus, highlighting the neurovascular, cellular, and molecular mechanisms linked to post-COVID-19 fatigue, this narrative review focuses on the molecular consequences of dipeptidyl peptidase IV (DPPIV). DPPIV’s putative role as a co-receptor for SARS-CoV-2 has attracted a lot of attention. Specifically, DPPIV is an important regulator of diabetes, obesity, hypertension, inflammation, and glucose metabolism—all of which are linked to the chronic cerebrovascular dysfunctions that follow COVID-19.

## 2. SARS-CoV-2, Post-COVID-19 Fatigue, and Multi-Organ Injury

The coronavirus is described as a glycoprotein-based surface spiked positive-stranded RNA virus enclosed in an envelope. It is a member of a large family of viruses that have long been present in both humans and animals around the globe. These viruses can cause everything from minor respiratory ailments to serious respiratory diseases. Of these, the following seven distinct coronavirus types have been found in humans: Middle East respiratory syndrome coronavirus (MERS-CoV), severe acute respiratory syndrome coronavirus (SARS-CoV), and human coronaviruses (HCoV) including HCoV-OC43, HCoV-229E, HKU1, and HCoV-NL63 [21]. These variants stand out due to their high pathogenicity and transmissibility. Moreover, SARS-CoV-2 is the sixth coronavirus reported to infect people. It is genetically identical to SARS-CoV and MERS-CoV, which are also present in bats. The neurological symptoms of the three major SARS-CoV, MERS-CoV, and SARS-CoV-2 outbreaks that resulted in worldwide pandemics show both variances and commonalities [21,22,23]. Moreover, SARS-CoV, which was discovered in 2002, and MERS-CoV, which was discovered in 2011, share sequence similarities according to recent research using the next-generation sequencing of the SARS-CoV-2 genome [24,25].

Even though most people infected with COVID-19 eventually recover, a consider-able percentage of people in different age groups experience several symptoms, including post-exertional malaise, for a lengthy time [1,2,3,4,5,6,7,8,9]. Recent findings indicate that an unbalanced autoimmune reaction and persistent virus particles in the body could cause these post-COVID-19 fatigue symptoms. Studies have shown that the virus is present in several tissues, including the brain [10,11]. Moreover, post-COVID-19 fatigue symptoms are similarly persistent in those with chronic autoimmune diseases [10,26].

Thus, the goal of the ongoing study is to understand the origins and consequences of SARS-CoV-2 infection, with a focus on the long-term effects on various organs and tissues. COVID-19 was first identified as a respiratory disease, but it has now been linked to multiple organ failures and neurological disorders [27,28,29]. In this case, the viral infection in the lungs triggers responses that can eventually develop into a systemic event impacting several organs, such as the heart and brain, and different hematologic abnormalities, regardless of the disease’s original severity. A slow and progressive failure of the gastrointestinal, endocrine, hepatic, renal, and circulatory systems may result from this trend [27,28,29,30]. The multi-organ injury and sequelae symptoms in post-COVID-19 fatigue are summarized in Table 1.

### Heart and Brain Crosstalk: Roles of Thrombo-Inflammation and Post-COVID-19 Fatigue

As COVID-19 symptoms persist, manifestations affecting the cardio-cerebrovascular systems are increasingly apparent. COVID-19 affects both large and small blood vasculature, raising the risk of thrombosis, and has a major effect on the coagulation cascade, hence leading to thrombo-inflammation. Over time, these problems may result in increased rates of morbidity and mortality from cardio-cerebrovascular diseases [30,42]. According to a recent study by the National Institute of Health and other studies, it was reported that the coronavirus can travel through systemic microcirculation to the brain and heart within a few days of infection and can stay there for a long time. Furthermore, research has shown that micro-clots can form in smaller blood vessels, which can obstruct cerebral blood flow. About 20% to 30% of COVID-19 patients who are sick have these clots [43,44].

There is evidence that SARS-CoV-2 is present in the brain’s hypothalamus and cerebrospinal fluid of COVID-19 patients. It is thought that the remaining virus population causes vascular inflammation, which results in symptoms related to cerebrovascular accidents. Reports of ischemic strokes, intracranial bleeding, and cerebral venous thrombosis have all been connected to the COVID-19-related clotting condition [31,45]. Uncontrolled immunological and inflammatory responses, which mediate hypercoagulability, endothelial dysfunction, and thrombosis, most likely start the clotting process. These processes may result in a rise in heart attacks, strokes, and deaths [32,46]. It is interesting to note that neuro-invasion and cerebrovascular symptoms are present in about two-thirds of post-COVID-19 fatigue patients. Headache, taste and smell abnormalities, encephalopathy, ischemic stroke, Guillain–Barré syndrome, cognitive impairment, dizziness, fast heartbeat, post-traumatic stress disorder, mini-strokes, anxiety, depression, and other mental health conditions are some of these symptoms [31,32,33,34,35,36,37]. Therefore, it is conceivable that the harm caused by COVID-19 to the central nervous system (CNS) and various organs may be progressive, necessitating extended periods for the recovery and restoration of normal activities [40,41].

## 3. Current Updates on Molecular and Cellular Mechanism of Post-COVID-19 Fatigue

Although the involvement of multiple microstructural (i.e., cellular) and molecular components in SARS-CoV-2 infection is well-known, the exact processes underlying post-COVID-19 fatigue are still vague. Extensive studies have been conducted to identify the complex pathways associated with post-COVID-19 fatigue. It is well-reported that the coronavirus attaches itself to the cell surface during the first stage of infection, acting as a portal for further infection. The vulnerability of a cell to viral infection is determined by the presence of receptors and cofactors on the cell surface. On the other hand, COVID-19 primarily targets cells in the kidneys, heart, lungs, immune system, and brain’s neuronal and glial cells. However, the digestive epithelium offers a different entrance point for SARS-CoV-2, which results in gastrointestinal symptoms. This may lead to the disruption of the intestinal barrier and systemic inflammation, which could impact the function of the CNS and the enteric nervous system [47,48]. Hence, post-COVID-19 fatigue may be linked to a variety of microstructural alterations or determinants, molecular effects, meso-structural reactions, and symptoms.

### 3.1. Microstructural Changes—Endothelial Cells (ECs)

Endothelial cells (ECs) are essential for maintaining barrier integrity, restricting the entry of circulating inflammatory cells and chemicals and regulating vascular tone under normal physiological conditions. The lining of blood vessels, such as arteries, veins, capillaries, and lymphatic vessels, is made up of ECs, which come into direct touch with virus particles and blood in circulation. ECs also produce several factors and enzymes that control blood coagulation, platelet adhesion, immunological response, and vascular relaxation and contraction [49]. ECs in blood arteries play a critical role in SARS-CoV-2 infection, causing damage, advancing COVID-19 illness, and perhaps spreading the infection to other organs. Increased thrombotic and inflammatory processes are intimately associated with microvascular EC dysfunction and have been connected to the symptoms of post-COVID-19 fatigue [50].

Angiotensin-converting enzyme 2 (ACE2) receptors are expressed on the surface of ECs, which allows SARS-CoV-2 to enter through its high-affinity spike protein. Endothelial injury, malfunction, and blood vessel damage result from this interaction. As a result, cytokines, chemokines, P-selectin, E-selectin, and intercellular adhesion molecule 1 (ICAM-1) are created, and these adhesion molecules draw different cells, including platelets, to the artery walls. Aggregation, coagulopathy, and a higher frequency of thrombotic events are all encouraged by this. Moreover, vasculitis is triggered by SARS-CoV-2 infection in the heart, which also causes dysfunction and chronic inflammation. Following EC dysfunction, SARS-CoV-2 virus particles in the brain may lead to blood–brain barrier (BBB) damage, triggering an innate immune reaction and escalating inflammation within brain microvascular ECs [50,51].

### 3.2. Microstructural Changes—Neuro–Glia–Vascular Unit (NGVU)

Recently, it has been well-documented that CNS cells are involved in COVID-19 infection. It has been discovered that SARS-CoV-2 directly infects human peripheral sensory neurons through interaction with the ACE2 receptor, leading to deficits in chemosensory perception [47]. Furthermore, COVID-19 has been linked, particularly in the hippocampus region of the brain, to neuronal degeneration and a reduction in neurogenesis. These results raise the possibility of memory impairment and long-term neurological problems, possibly through oxidative stress and neuro-inflammation processes [52]. Additionally, there is a recognized regulatory role that this connection between ECs and the NGVU plays in cerebrovascular disorders. Research has indicated that SARS-CoV-2 causes hypoxic brain alterations that impact the cerebellum and cerebrum, among other brain regions. As a result, this causes the death of neurons in important regions such as the hippocampus, cerebral cortex, and the cerebellum’s Purkinje cell layer [53].

The systemic inflammatory reactions that begin in the peripheral areas may be the cause of the CNS issues seen in post-COVID-19 fatigue patients. Furthermore, viral infection activates glial cells, including microglia and astrocytes, which may increase the vulnerability to neurodegenerative illnesses [54]. According to recent studies, the SARS-CoV-2 infection causes an inflammatory response that activates both astrocytic and microglial processes [55]. These processes are linked to aberrant phenotypes in the interactions between astrocytes and the vasculature, which are essential for preserving the BBB integrity. Peripheral cell infiltration into the brain is facilitated in COVID-19 patients by an impaired BBB. Moreover, even in the absence of overt clinical neurological symptoms, increased levels of secreted neural and glial proteins, such as serum neurofilament (sNfL) and serum glial fibrillary acidic protein (sGFAP), suggest neuro–glial degeneration in people with COVID-19 [56,57].

### 3.3. Meso-Structural Responses—Pro-Inflammatory Cells

It has been shown that the SARS-CoV-2-infection-induced dysregulated host inflammatory response exacerbates hypercoagulability, EC dysfunction, and thrombosis. These variables exacerbate cerebrovascular disease, which raises the risk of stroke and its associated mortality [58]. Previous studies have extensively described the complex and distinct cellular immune response during acute, resolving, or progressive COVID-19. Activated immune cells have been found in the blood of COVID-19 patients for a significant amount of time, from one to eight months after infection [59]. Research has demonstrated the involvement of monocytes and different subtypes of macrophages in the lung, which is correlated with outcomes in COVID-19-affected people [60]. Furthermore, lymphocytes, which are made up of B cells, T cells, and natural killer cells, are other vital immune components. Studies have also shown that those with post-COVID-19 fatigue had different T cell characteristics, such as worn-out T cells and fewer CD4+ and CD8+ effector memory cells [61,62].

### 3.4. Molecular Mechanism of Post-COVID-19 Fatigue

Understanding the molecular processes and feedback loops that underlie COVID-19 is essential for developing treatment strategies that will lessen the disease’s long-term effects. Through its interactions with the genome, the virus modifies the intricate structures of proteins and DNA, changing gene expression and causing immunological dysregulation. Since these molecular changes may have long-term effects, it is critical to comprehend and target them in therapeutic interventions [63].

An important biological mechanism linked to increased illness severity and persistence is the overproduction of pro-inflammatory cytokines (or cytokine storms) caused by the virus. These include interferon (IFN)-γ, IFN-γ-inducible protein 10, monocyte chemoattractant protein-1 (MCP-1), granulocyte colony-stimulating factor (GCSF), macrophage inflammatory protein-1 alpha (MIP-1α), and tumor necrotic factor alpha (TNF-α), in addition to interleukins such IL-1β, IL-2, IL-6, IL-7, IL-8, IL-10, and IL-17 [64,65]. When the transcription factor such as nuclear factor kappa B (NF-κB) is activated, cytokines and chemokines are produced, which attract and activate immune cells from the circulation. An uncontrolled cytokine storm worsens organ failure, vascular permeability, adhesion characteristics, pro-coagulation of blood, and COVID-19-mediated inflammation and oxidative stress, along with long-lasting tissue damage. Higher blood biomarkers of the vascular system in post-COVID-19 fatigue patients suggest that angiogenesis-regulating molecules are important components of this pathophysiological mechanism [66,67].

Additional molecular biomarkers linked to COVID-19 comprise pro-calcitonin, phospholipids, D-dimer, serum ferritin, and C-reactive protein. These biomarkers function as indications of severe organ failure and are implicated in the production of micro-clots. Furthermore, the increased expression of surface proteins that indicate endothelial dysfunction includes E-selectin, vascular cell adhesion molecule 1 (VCAM-1), and ICAM-1. These molecular factors have the potential to worsen issues in the neurological, cardiovascular, gastrointestinal, liver, and kidney systems [68,69]. Thus, the identification of the post-COVID-19 fatigue prevalence and the molecular factors that underlie it has great potential for identifying therapeutic targets that will either stop the illness from starting or lessen its symptoms. Through a comprehensive comprehension of the distinct biochemical pathways implicated in post-COVID-19 fatigue, scholars might identify possible therapies aimed at attenuating its impact, thereby providing alleviation to individuals enduring symptoms.

On the other hand, the first phase of the infectious viral life cycle is represented by the recognition of and interactions with cellular receptors. It is a well-known fact that the SARS-CoV-2 virus binds to the ACE2 receptor, which is located on the surface of cells in the digestive and respiratory systems, to begin the infection process [70]. However, DPPIV, neuropilin-1 (NRP1), and transmembrane serine protease type 2 (TMPRSS2) are other molecules that have been discovered as possible targets for the virus [71]. The expression of several molecular receptors on the cell surface could therefore be connected to different routes of viral entry for SARS-CoV-2 [71]. Moreover, DPPIV offers unique insights that distinguish it from the ACE2 pathway, particularly in its broader involvement in metabolic and inflammatory pathways. Unlike ACE2, which is mainly linked to direct viral entry, DPPIV is deeply involved in regulating glucose metabolism, immune responses, and chronic inflammation, all of which are key in the progression of post-COVID-19 fatigue and cerebrovascular complications [72]. The most common comorbidities seen in COVID-19 patients were immune system, cardiovascular, neurological, and diabetic diseases, as well as hypertension, atherosclerosis, and other conditions. Surprisingly, type 2 diabetes mellitus (T2DM) is linked to DPPIV in these illnesses. Further investigation into how DPPIV affects COVID-19 intensity and the length of persistent symptoms is, therefore, necessary [73,74,75].

Furthermore, DPPIV’s enzymatic role in degrading glucagon-like peptide-1 (GLP-1) links it to hyperglycemia, a significant risk factor for severe COVID-19 outcomes [74]. This hyperglycemic state, often exacerbated in patients with metabolic disorders such as diabetes, fuels systemic inflammation and endothelial dysfunction, which increases the risk of microvascular damage in the brain [76]. Unlike ACE2, DPPIV not only facilitates viral entry, but also amplifies the metabolic and inflammatory disruptions that contribute to the long-term neurovascular damage seen in post-COVID-19 patients.

Thus, while ACE2 primarily acts as the gateway for SARS-CoV-2 infection, DPPIV may putatively serve as both a co-receptor and a key mediator of the metabolic and inflammatory cascades that aggravate the course of infection [77,78]. This dual role makes DPPIV a potential therapeutic target not just for limiting viral entry, but for managing the chronic complications, such as stroke and cognitive decline, that are linked to metabolic and cerebrovascular dysregulation in COVID-19 survivors.

## 4. Novel Insights into DPPIV’s Role in Metabolic Syndrome and COVID-19

**Figure 1:**
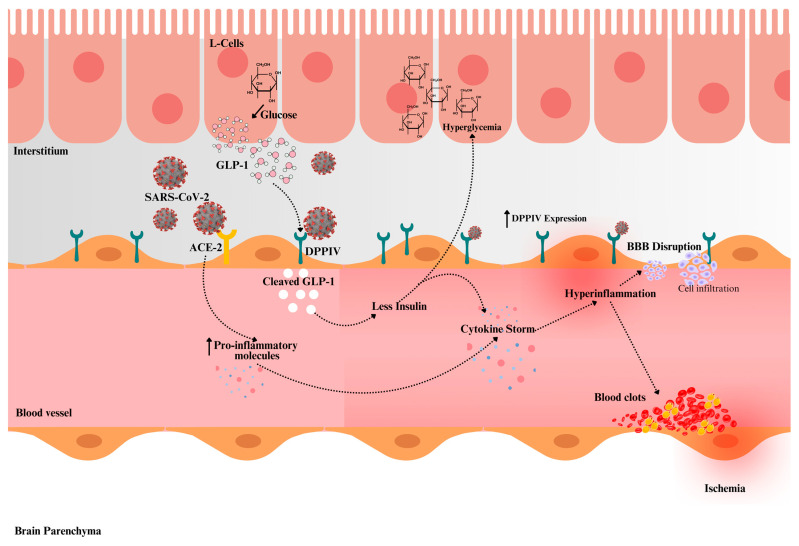
Proposed mechanism illustrating DPPIV-mediated SARS-CoV-2 infection and its impact on glucose metabolism and cerebrovascular health. DPPIV degrades GLP-1 in the bloodstream, leading to reduced insulin secretion and hyperglycemia. This hyperglycemic state, along with the cytokine storm triggered by SARS-CoV-2, exacerbates endothelial dysfunction and inflammation, resulting in blood–brain barrier (BBB) disruption, cell infiltration, blood clot and microthrombus formation, and increased risk of cerebrovascular complications, including stroke. Darker red in the blood vessel structure indicates an inflamed vessel. The upward arrow indicates “increase”.

The course and results of COVID-19 are considerably impacted by metabolic diseases such as T2DM [74]. Chemokines and their receptors have been identified as a shared pathway between T2DM and COVID-19 by an investigation of pathway connections. By controlling the functions of chemokines, glucose homeostasis, and metabolism, DPPIV is essential in T2DM [74]. Notably, there is a substantial correlation between hyperglycemia and diabetes and elevated DPPIV activity. It is well-known that DPPIV causes disruptions in glucose metabolism by degrading active GLP-1, which raises blood glucose levels [76]. As illustrated in Figure 1, DPPIV’s enzymatic activity reduces GLP-1 levels, exacerbating hyperglycemia, which, alongside the SARS-CoV-2-induced cytokine storm, leads to endothelial dysfunction, increased microthrombi, and heightened cerebrovascular risks.

It is conceivable that metabolic syndrome associated with comorbidities including obesity, diabetes, and cerebrovascular disorders is related to hyperglycemia, which may promote viral shedding and reproduction. This could, therefore, worsen COVID-19-related inflammation and cause endothelial dysfunction. Moreover, a hyperglycemic state is brought on by the multi-systemic inflammation associated with SARS-CoV-2 infection; on the other hand, this hyperglycemic state may facilitate viral replication [79]. The uncontrolled glycosylation of SARS-CoV-2 modifies viral epitopes, facilitating the evasion of immune system detection. This evasion leads to a more severe and prolonged COVID-19 infection in hyperglycemic diabetic individuals [80].

Moreover, many physiological systems, such as the metabolism, obesity, autoimmunity, the immune system, endocrine functions, inflammation, cellular activities, and thrombotic cerebrovascular disorders like stroke, are known to be regulated by the adaptable transmembrane and circulating protein DPPIV [81]. Numerous cell types, including T cells, B cells, NK cells, certain macrophage subsets, hematopoietic stem cells, and hematopoietic progenitor cells, have a broad expression of this protein. It can also be found on the surface of acinar, endothelial, and bone marrow cells in a variety of organs, including the kidney, liver, intestines, lung, spleen, and pancreas [81,82]. Moreover, when DPPIV is enzymatically active, it behaves as a homodimer, whereby numerous cytokines, including interferons, hepatocyte NF-1α, and hypoxia-inducible factor-1α, can strongly stimulate its expression. Pro-inflammatory diseases like obesity, diabetes, and atherosclerosis, and cerebral and cardiovascular disorders like stroke are associated with markedly elevated DPPIV levels [81,82,83]. Through its effects on inflammatory signaling pathways, stimulation of vascular smooth cell proliferation, and induction of oxidative stress in various cell types, imbalanced levels of DPPIV may make these pathological states worse. Alarmingly, DPPIV’s immunomodulatory effects can cause inflammation on both a local and systemic level. It has been shown that DPPIV may promote T cell activation, proliferation, and signal transduction, among other aspects of lymphocyte function. Moreover, after an acute infection, DPPIV is likely involved in a new regulatory mechanism that suppresses the immune response against the SARS-CoV-2 virus [84].

### 4.1. Potential Role of DPPIV as Receptor for SARS-CoV-2 Infection

In addition to controlling inflammation and hyperglycemia, DPPIV has recently attracted attention concerning SARS-CoV-2 infection because it may act as a co-receptor for the virus. Remarkably, DPPIV has been found to function as a co-receptor for the entry of some viruses, such as the coronavirus MERS-CoV and the human immunodeficiency virus (HIV) [85]. Studies reveal that DPPIV’s critical binding sites required for this interaction coincide with those that MERS-CoV-S binds to. Additionally, it has been established that DPPIV serves as a useful receptor for cellular invasion when it comes to Human Coronavirus-Erasmus Medical Center (hCoV-EMC). Antibodies directed against DPP4 did, in fact, successfully prevent hCoV-EMC infection in Huh-7 and human bronchial epithelial cells [85,86,87].

Moreover, protein–protein docking experiments using crystal structures have demonstrated a strong affinity between DPPIV and the spike-receptor-binding domain of SARS-CoV-2, which is particularly noteworthy [88]. Furthermore, silico computational studies have indicated DPPIV as a likely SARS-CoV-2 receptor. Building on these discoveries and hypotheses, a growing body of research suggests that DPPIV might serve as a functional co-receptor for human coronaviruses, hence aiding in the transmission of SARS-CoV-2 [88,89,90,91].

### 4.2. Emerging Research on DPPIV and COVID-19

In this context, DPPIV has been analyzed in COVID-19 studies ranging from pre-clinical, including animal models and in vitro studies, to clinical and observational ones. These collectively provide important insights into how DPPIV influences disease progression, with particular emphasis on inflammation, the glucose metabolism, and cerebrovascular outcomes.

Pre-clinical animal studies, including its involvement in COVID-19, have investigated the involvement of DPPIV during viral infections. Evidence from recent animal models indicated that DPPIV inhibition led to lower inflammation and neurovascular damage attributed to COVID-19-like symptoms [92]. These findings point out the enzyme’s contribution to the inflammation process, particularly in exacerbating the cytokine storm leading to endothelial dysfunction and cerebrovascular complications.

For instance, recent findings have identified that the inhibition of DPPIV in animal models could mitigate SARS-CoV-2-induced endothelial injury in the brain, reducing the incidence of microthrombi [93,94]. Such a model has, until now, helped establish the role of the enzyme in increasing COVID-19-related cerebrovascular risks, especially in aged and diabetic animal models. DPPIV inhibitors such as gliptins (i.e., saxagliptin, alogliptin sitagliptin, and linagliptin) have also been a focus of various clinical studies concerning COVID-19 patients, specifically those with T2DM. They are said to decrease the severity of COVID-19 through the limited viral entry and modulation of the immune response [95,96]. Additionally, diabetic patients receiving DPPIV inhibitors had lower viral loads, reduced levels of inflammatory markers, and a lower incidence of cerebrovascular complications compared to controls [97].

Observational studies further stress the high activity of DPPIV with the poor outcomes in COVID-19 disease. For example, elderly COVID-19 patients reported that higher levels of DPPIV were related to increased cerebrovascular complications such as stroke and cognitive decline [98,99]. Such observations have shown that DPPIV acts as a potential biomarker for the anticipation of cerebrovascular risks among post-COVID-19 patients, particularly among patients with comorbid conditions like diabetes and hypertension.

All in all, the studies presented highlight DPPIV’s central role in modulating COVID-19 severity, particularly through its effects on inflammation, immune response, and glucose metabolism. In animal models, DPPIV inhibition has shown promise in reducing the impact of COVID-19 on the brain and cardiovascular system. Meanwhile, clinical trials suggest that DPPIV inhibitors may offer therapeutic potential, especially for diabetic patients who are more prone to severe outcomes. Observational studies further support the enzyme’s involvement in post-COVID-19 cerebrovascular complications.

## 5. DDPIV and Post-COVID-19 Fatigue: Impact on Cerebrovascular Disease

Vascular disorders, including embolisms and thrombotic cerebrovascular diseases, as well as cardiovascular diseases, hypertension, diabetes, and obesity, are the main comorbidities linked to post-COVID-19 fatigue. Severe COVID-19 cases can cause vascular integrity to be disrupted, coagulation and inflammatory responses to be triggered, and atherosclerosis to be encouraged, all of which increase the risk of cerebrovascular disorders. The virus can also harm the structure and function of the brain, which can lead to long-term cognitive impairment.

Alarmingly, those with COVID-19 who have abnormalities in their glucose metabolism are more likely to die from vascular–neurological issues and to experience other COVID-19-related complications down the road. This could put them at risk for COVID-19’s long-term repercussions. Thus, DPPIV, i.e., a vascular system component that affects glucose metabolism, is essential in controlling these disorders. A recent study has shown that DPPIV is dysregulated in the brain after a stroke. Through an in vitro functional study, recent reports have discovered that DPPIV inhibits the migration of neural progenitor cells and decreases the angiogenic capacity of brain ECs mediated by stromal cell-derived factor 1 (SDF1). The processes of tissue remodeling and repair in the brain following a stroke may be hampered by the overexpression of DPPIV in response to ischemia/reperfusion injury [100]. Table 2 summarizes the role of DPPIV in post-COVID-19 cerebrovascular complications.

DPPIV plays a multifaceted role in the pathophysiology of COVID-19, particularly concerning cerebrovascular complications. Its ability to exacerbate endothelial dysfunction, promote inflammation, and disrupt glucose metabolism highlights the enzyme’s critical role in increasing the risk of post-COVID-19 cerebrovascular events. As such, DPPIV may represent both a key biomarker for predicting cerebrovascular complications and a potential therapeutic target for mitigating these risks in high-risk COVID-19 patients, especially those with metabolic comorbidities. While the exact mechanisms of DPPIV’s involvement in SARS-CoV-2 infection are still being explored, the existing evidence suggests that targeting DPPIV may reduce the risk of long-term cerebrovascular complications, including stroke and cognitive decline, in post-COVID-19 patients. Future research should continue to explore the therapeutic potential of DPPIV inhibitors in reducing both acute and chronic COVID-19 outcomes.

Studies have shown that DPPIV inhibitors may protect against stroke and vascular illnesses. Research on DPPIV, both scientific and clinical, has drawn interest not just in diabetes but also in cerebrovascular disorders. Thus, it is possible that increased DPPIV levels, which support hyperglycemia and increased inflammation brought on by cytokine storms, are connected to the long-term neurological problems and vascular cognitive impairment linked to COVID-19. DPPIV has been identified as a potential factor aggravating inflammation and the long-term effects of viral infections, according to the research evaluated in this context. Consequently, using DPPIV inhibitors in COVID-19 patients may offer a simple method of reducing viral entrance and replication, even if the patient has diabetes. This strategy may provide efficient therapeutic approaches to counteract inflammation and metabolic disruptions brought on by COVID-19. However, a more thorough investigation is required to clarify DPPIV’s role in post-COVID-19 fatigue.

### 5.1. Confounding Factors Influencing DPPIV-Mediated Post-COVID-19 Fatigue and Cerebrovascular Complications

Age, genetic predisposition, and the presence of comorbidities, including diabetes, hypertension, and obesity, may further modulate the severity of fatigue and cerebrovascular complications post-COVID-19 through the interaction with DPPIV activity [101]. Indeed, it has been documented that these comorbid conditions modulate the levels and activities of DPPIV, leading to exacerbated inflammation and metabolic pathways that drive severe outcomes in COVID-19 patients.

Aging is associated with a natural increase in DPPIV activity [102], which, in the cases of elderly patients affected by COVID-19, may worsen the outcome. According to the obtained data, elderly patients exhibit increased circulating levels of DPPIV, which are associated with an increased inflammatory response and a higher risk of developing cerebrovascular complications [74]. For example, one multicenter cohort study reported that, among elderly COVID-19 patients, high levels of DPPIV were associated with a higher incidence of stroke and cognitive decline, probably as a result of increased endothelial dysfunction and microthrombus formation [103]. This age-dependent increase in the enzymatic activity of DPPIV may further potentiate the cytokine storm observed in severe COVID-19 and thus contribute to post-COVID-19 fatigue and neurovascular damage. Thus, the increased inflammation burden in elderly subjects, along with high levels of DPPIV, places them at greater risk for neurological complications long after SARS-CoV-2 infection.

Moreover, metabolic disorders such as T2DM, obesity, and hypertension are directly associated with increased DPPIV activity, which, in turn, is associated with the severity of COVID-19 and its complications [74]. DPPIV has an important role in glucose metabolism; patients with T2DM have increased levels of DPPIV due to GLP-1 degradation [74]. This leads to hyperglycemia, considered a risk factor for poor outcomes in COVID-19. Moreover, in diabetic or obese patients, the hyperglycemic environment mediates viral replication and systemic inflammation, increasing the risks of cerebrovascular complications such as stroke and cognitive impairment [104,105]. Very recent studies also reported that COVID-19 diabetic patients with high DPPIV levels had more severe cerebrovascular events compared to non-diabetic COVID-19 patients, further underlining the pivotal role this enzyme plays in bridging metabolic dysfunction to COVID-19 severity [106].

This was also true for hypertension, another frequent comorbidity in COVID-19 patients, which presented higher levels of DPPIV [74]. Perhaps through the exacerbation of endothelial dysfunction and inflammation, known comorbid conditions that may be particularly exacerbated by the presence of DPPIV, individuals with hypertension would indeed suffer worse cerebrovascular outcomes. Moreover, genetic predispositions related to single nucleotide polymorphisms in the gene encoding DPPIV or related pathways could be relevant in determining the poor outcomes of some individuals affected by COVID-19 [107]. Although direct studies relating genetic predisposition to DPPIV-mediated COVID-19 complications are still emerging, there is evidence to date that polymorphisms in the gene encoding DPPIV influence its expression and activity [76]. These genetic variations may modulate the predisposition of an individual to the neurovascular and metabolic complications associated with COVID-19.

Moreover, genetic factors related to immune response and inflammation may interact with DPPIV activity. For example, individuals with a predisposition to heightened inflammatory responses may experience a more severe cytokine storm when combined with elevated DPPIV activity, leading to prolonged post-COVID-19 fatigue and cerebrovascular dysfunction. Table 3 summarizes the confounding factors influencing DPPIV-mediated COVID-19 outcomes.

These confounding factors highlight the multifaceted nature of DPPIV’s influence on COVID-19 severity, particularly in post-COVID-19 fatigue and cerebrovascular complications. Age-related increases in DPPIV activity, combined with comorbidities such as diabetes and hypertension, significantly exacerbate the inflammatory and metabolic disruptions caused by COVID-19. Future research should focus on understanding the genetic predispositions that may further modulate DPPIV activity and how these factors collectively contribute to long-term COVID-19 sequelae.

### 5.2. Future Recommendations

Post-COVID-19 fatigue presents a multifaceted challenge, characterized by a diverse array of symptoms that can manifest regardless of the initial severity of the disease. Since these features of post-COVID-19 fatigue are complex and multifactorial, research and clinical practices should aim at the identification of specific biomarkers of early diagnosis, which may allow clinicians to better predict symptom trajectories and timely management. The primary focus in addressing post-COVID-19 fatigue lies in comprehending its progression and identifying effective treatment strategies, i.e., in this case, focusing on DPPIV. Researchers are beginning to delineate the specific clinical, cellular, and molecular mechanisms underlying post-COVID-19 fatigue symptoms and DPPIV. Neurovascular disorders may play a role in driving the progression of post-COVID-19 fatigue, with SARS-CoV-2 infection and the role of DPPIV as a co-receptor potentially causing tissue and cellular damage, as well as exacerbating inflammatory or autoimmune responses due to disruptions in the proteolytic enzyme activities, catalysis, and molecular signaling pathways.

While existing research has contributed to our understanding of the mechanisms underlying post-COVID-19 fatigue, numerous unanswered questions persist and warrant urgent attention to mitigate its effects. Identifying additional molecular determinants and target proteins, i.e., DPPIV, is crucial for alleviating the substantial burden of post-COVID-19 fatigue. Applying advanced molecular profiling in searching for the singular biomarker of post-COVID-19 fatigue and neurovascular dysfunction, specifically those related to DPPIV and other proteolytic enzyme pathways by using techniques such as single-cell RNA sequencing, metabolomics, proteomics, whole-genome sequencing, and gut microbiome studies offer promising avenues to unravel the pathophysiological processes underlying post-COVID-19 fatigue.

Further extension of pharmacological research into DPPIV inhibitors and modulators of neuroinflammatory responses might allow for targeted treatments that could alleviate symptoms directly related to immune and inflammatory dysregulation. This may minimize neuroinflammatory effects promoting fatigue and possibly reduce the risk of chronic neurovascular complications in these patients. In addition, such a study on the interplay between the gut microbiome and neurovascular health in post-COVID-19 fatigue is clinically important because there is emerging evidence that dysbiosis of the microbiome might be associated with immune and neurological dysfunction.

Longitudinal clinical trials should also be a focus of investigation in the assessment of the long-term effects of SARS-CoV-2 infection on neurovascular and cognitive health. Such trials would allow the identification of at-risk individuals, helping clinicians tailor preventative and therapeutic interventions, especially in those likely to develop chronic cerebrovascular conditions. Integration of assessment for cognitive function, neuroimaging biomarkers, and vascular health over extended follow-ups can give robust insights into disease progression and intervention efficacy.

The integration of data from basic science and clinical research is essential for gaining insight into the viral invasion into various tissues, particularly the brain, where it leads to neurological dysfunctions. Developing improved therapies to mitigate long-term cerebrovascular disease incidence and progression resulting from SARS-CoV-2 infection hinges on these investigations. Finally, further extensive research is undoubtedly necessary to evaluate and devise targeted treatment strategies for combating the persistent effects of post-COVID-19 fatigue.

#### Limitations of Current Narrative Review

We acknowledge that such a narrative approach has its limitations, including selection bias and the lack of quantitative synthesis. These limitations are partly offset by the comprehensiveness and richness of our expert analysis, which provides a very strong foundation for understanding neurological sequelae following post-COVID-19 fatigue. When the body of research builds up, a future systematic review could analyze the well-defined studies to quantify the impact and underlying mechanisms of DPPIV-mediated cerebrovascular complications. A review might consider extensive inclusion criteria and data analysis to define more precise relationships. We strongly believe that our narrative review epitomizes the current knowledge on the subject and thus lays a foundation for further systematic studies in the future as the body of research grows.

## 6. Conclusions

In conclusion, from this review, the immense potential of DPPIV has become clear not only as a co-receptor of SARS-CoV-2 but also as a main mediator in metabolic–inflammatory cascades leading to the aggravation of post-COVID-19 fatigue and cerebrovascular complications. Its participation in glucose metabolism, immune regulation, and endothelial dysfunction, therefore, places it at the heart of the chronic cerebrovascular outcomes that accompany COVID-19 survivors, such as neurovascular damage, stroke, and cognitive decline. Targeting DPPIV with therapeutic inhibitors holds immense promise toward the mitigation of long-term cardiovascular and cerebrovascular risks, particularly in post-COVID-19 fatigue patients with comorbidities that include diabetes and hypertension. Further studies are necessary to demonstrate the exact molecular mechanisms and establish the therapeutic value of DPPIV inhibitors in the management of post-COVID-19 fatigue as an effective way to reduce the public health burden of this debilitating condition.

## Figures and Tables

**Table 1:** Multi-organ injury and sequelae symptoms in post-COVID-19 fatigue.

Organ/System.	Sequel Symptoms	References
Nervous System	Brain fog; cognitive and memory	[31,32,33,34,35,36,37]
	Dizziness	
	Headache	
	Stroke	
	Joint pain	
	EC dysfunction—BBB damage	
	Microthromboembolism	
	Anxiety, stress, and depression	
	Anosmia (loss of sense of smell)	
	Ageusia (loss of taste)	
	Insomnia (sleep disturbance)	
	Guillain–Barré syndrome	
	Chronic fatigue	
	Encephalopathy	
Respiratory	Cough	[4,5]
	Sore throat	
	Dyspnea (difficulty of breathing)	
	Modified diffusion capacity restricting pattern, and obstructive pattern	
Cardiovascular	Myocardial damage	[38]
	Palpitations	
	An irregular pulse	
	Chest pain	
	Myocarditis	
Gastrointestinal	Diarrhea	[39,40,41]
	Vomiting/nausea	

EC, endothelial cell; BBB, blood–brain barrier.

**Table 2:** Summary of DPPIV’s role in post-COVID-19 cerebrovascular complication.

Mechanism	DPPIV Role	COVID-19-Related Cerebrovascular Complications
Endothelial dysfunction	DPPIV degrades GLP-1, leading to endothelial damage and impaired vascular function	Increases risk of stroke, microthrombi, and blood–brain barrier disruption
Inflammatory pathways	DPPIV activates pro-inflammatory cytokines (TNF-α, IL-6, and IL-1β), enhancing immune response	Exacerbates vascular inflammation and thrombotic events
Glucose metabolism	DPPIV’s regulation of glucose homeostasis is disrupted, particularly in diabetic patients	Hyperglycemia worsens inflammation and thrombosis, leading to stroke
Therapeutic inhibition	DPPIV inhibitors (gliptins) reduce inflammation, endothelial dysfunction, and hyperglycemia	Potential to lower cerebrovascular risks in COVID-19 patients

DDPIV, dipeptidyl peptidase IV; GLP-1, glucagon-like peptide-1; ILs, interleukins; TNF-α, tumor necrosis factor-alpha.

**Table 3:** Summary of confounding factors influencing DPPIV-mediated COVID-19 outcomes.

Confounding Factor	Impact on DPPIV Activity	Influence on Post-COVID-19 Complications
Age	Increases with age, leading to elevated inflammation and endothelial dysfunction	Heightened risk of stroke, cognitive decline, and post-COVID-19 fatigue
Diabetes and obesity	Elevated DPPIV levels due to glucose metabolism dysregulation	Worsens hyperglycemia and inflammation, and increases cerebrovascular risks
Hypertension	Increased DPPIV linked to worsened endothelial dysfunction and inflammation	Greater risk of cerebrovascular complications and neurovascular damage
Genetic predisposition	Genetic variations may influence DPPIV expression and activity	May heighten susceptibility to cerebrovascular and inflammatory complications
